# The sympathetic nervous system in heart failure with preserved ejection fraction

**DOI:** 10.1007/s10741-024-10456-0

**Published:** 2024-10-23

**Authors:** Joshua W.-H. Chang, Rohit Ramchandra

**Affiliations:** Manaaki Manawa – The Centre for Heart Research, Department of Physiology, University of Auckland, Auckland, New Zealand

**Keywords:** Autonomic, Diastolic, Exercise, Heart failure, HFpEF, Sympathetic

## Abstract

The sympathetic nervous system (SNS) is a major mediator of cardiovascular physiology during exercise in healthy people. However, its role in heart failure with preserved ejection fraction (HFpEF), where exercise intolerance is a cardinal symptom, has remained relatively unexplored. The present review summarizes and critically explores the currently limited data on SNS changes in HFpEF patients with a particular emphasis on caveats of the data and the implications for its subsequent interpretation. While direct measurements of SNS activity in HFpEF patients is scarce, modest increases in resting levels of muscle sympathetic nerve activity are apparent, although this may be due to the co-morbidities associated with the syndrome rather than HFpEF per se. In addition, despite some evidence for dysfunctional sympathetic signaling in the heart, there is no clear evidence for elevated cardiac sympathetic nerve activity. The lack of a compelling prognostic benefit with use of β-blockers in HFpEF patients also suggests a lack of sympathetic hyperactivity to the heart. Similarly, while renal and splanchnic denervation studies have been performed in HFpEF patients, there is no concrete evidence that the sympathetic nerves innervating these organs exhibit heightened activity. Taken together, the totality of data suggests limited evidence for elevated sympathetic nerve activity in HFpEF and that any SNS perturbations that do occur are not universal to all HFpEF patients. Finally, how the SNS responds during exertion in HFpEF patients remains unknown and requires urgent investigation.

## Introduction

Heart failure with preserved ejection fraction (HFpEF) is a clinical syndrome in which symptoms and signs of heart failure (e.g., breathlessness, fluid retention, fatigue) occur despite a grossly normal left ventricular ejection fraction (LVEF ≥ 50%) [1]. HFpEF is now the most common form of heart failure and despite its poor prognosis and substantial socioeconomic burden, has few clinically impactful therapies [2, 3].

Universal findings in patients with HFpEF include elevated left ventricular filling pressure due to diastolic dysfunction and severe exercise intolerance with symptoms of breathlessness and fatigue during routine day-to-day activity with often normal resting function [4]. As the syndrome progresses, these symptoms become more apparent in patients with progressively lower levels of activity [2]. The pathophysiology of exercise intolerance in HFpEF is complex and incompletely understood but involves impairments in both cardiac (central) and extracardiac (peripheral) reserve capacity. These impairments include depressed chronotropic, inotropic, lusitropic, and vasodilatory reserves, which lead to attenuated increases in cardiac output during exercise, while the dysfunction of peripheral structures such as skeletal muscle also contributes to exercise intolerance in HFpEF [5].

In normal physiology, the sympathetic (and parasympathetic) nervous system mediates coordinated changes in cardiovascular variables in response to exercise [6], as well as fuel mobilization and hormone secretion [7]. Hence, it would be reasonable to hypothesize that perturbations of the sympathetic nervous system (SNS) contribute to HFpEF pathophysiology. Indeed, while many of the risk factors for HFpEF such as advanced age, hypertension, obesity, and type 2 diabetes mellitus are individually associated with chronic dysfunction of the SNS [8, 9], the role of the SNS in HFpEF has not been scrutinized in detail. In contrast, sympathetic overactivity has been relatively well-characterized in heart failure with reduced ejection fraction (HFrEF), which along with the well-documented contributions of enhanced activity from the renin–angiotensin–aldosterone system (RAAS), have formed the basis of modern therapy for HFrEF [10]. While previous reviews have covered the topic of the SNS in HFpEF [11–13], the purpose of the present review is (i) to provide a contemporary and comprehensive summary of the currently limited data on SNS activity in HFpEF patients and (ii) to emphasize the caveats with which the data were obtained as this has major implications for its interpretation. This latter point is particularly pertinent as definitive, but often inaccurate, conclusions about SNS activity are frequently encountered within the literature without detailed consideration of the context in which the data were obtained.

## The sympathetic nervous system in heart failure with preserved ejection fraction

The present review will begin by discussing “global” measures of sympathetic drive before focusing on sympathetic nerve activity to individual organs in HFpEF.

### Global activity of the sympathetic nervous system in HFpEF

Norepinephrine (NE) is the major post-ganglionic neurotransmitter of the SNS. A proportion of NE released by post-ganglionic sympathetic nerves diffuses (or spills over) from the synapse into the blood, escaping neuronal re-uptake and local metabolism [14, 15]. As a result, the amount of NE in the blood can be measured to serve as an index of sympathetic nerve activity. In this context, total plasma NE is frequently used to estimate global activity of the SNS.

Table 1 summarizes the studies that have reported total plasma NE in stable (compensated) HFpEF patients at rest. The consensus is that different studies have reported variable changes in plasma NE. Consequently, differing conclusions have been drawn. Some studies report no difference in elevated circulating NE levels between various heart failure groups based on ejection fraction [16–18]. In comparison, Nigmatullina et al. [19] found that heart failure patients with diastolic dysfunction and a normal ejection fraction (regardless of whether left ventricular hypertrophy was present or not) had higher levels of plasma NE than those with both diastolic and systolic dysfunction (reduced LVEF) whose plasma NE levels were normal when compared to healthy controls. This contrasts with findings by Aikawa et al. [20] who reported that circulating NE levels were similar amongst control participants and HFpEF patients that were stratified by severity of diastolic dysfunction. There is also discrepancy between HFrEF and HFpEF with some studies showing that the degree of sympathetic activation based on this measure is lower [21, 22] or normal [23, 24] in HFpEF patients compared to those with HFrEF.

**Table 1:** Summary of studies that have reported total plasma norepinephrine (NE) in HFpEF patients. Plasma NE measured in compensated primary HFpEF patients under resting conditions except where indicated

Study authors	Left ventricular ejection fraction (LVEF) threshold for HFpEF	Control group characteristics (if applicable)	Resting total plasma NE	Comments
Borlaug et al. [25]	≥ 50%	Matched for age, sex, comorbidities	Control = HFpEF	Total plasma NE in control = HFpEF at peak exercise
Yamaguchi et al. [83]	≥ 45%	Matched for age, sex, hypertension	Control = HFpEF	
Aikawa et al. [20, 55]	≥ 40%; uncertain if secondary HFpEF included	Matched for age but not sex	Control = HFpEF	Total plasma NE is similar regardless of severity of diastolic dysfunction [20] or presence or absence of coronary artery disease [55] in HFpEF
Benedict et al. [23]	> 45%; possible inclusion of secondary HFpEF	Matched for age but not sex	Control = HFpEF < HFrEF	
Seravalle et al. [24]	≥ 50%	Matched for age and sex	Control = HFpEF < HFrEF	
Jimenez-Marrero et al. [21]	≥ 50%		No control; HFpEF < HFrEF	
Vergaro et al. [22]	≥ 50%		No control; HFpEF < HFrEF; subset of HFpEF had values above laboratory upper limit of normal	
Patel et al. [28]	≥ 50%		No control; subset (12/25) of HFpEF had values above laboratory upper limit of normal	Total plasma NE unchanged by renal denervation
Kaye et al. [45]	> 50%	Matched for sex	Control < HFpEF	
Kitzman et al. [16]	≥ 50%	Matched for age but not sex	Control < HFpEF = HFrEF	
Vinch et al. [26]	≥ 50%; decompensated heart failure, possible inclusion of secondary HFpEF	Matched for age but not sex	Control < HFpEF = HFrEF	Total plasma NE unchanged when compensated
Tsuchida et al. [17]	≥ 45%; uncertain if secondary HFpEF included		No control; HFpEF = HFrEF	Total plasma NE in HFpEF = HFrEF at peak exercise
Wever-Pinzon and Fang [18]	≥ 50%; uncertain if secondary HFpEF included		No control; HFpEF = HFmrEF = HFrEF	
Nigmatullina et al. [19]	Not reported (mean LVEF = 59.7% and 56.5% in HFpEF groups); includes secondary HFpEF	Not reported in detail (aged 39–61 years, 85.7% male, healthy)	Control = HFrEF < HFpEF	

During exercise, total plasma NE rises in patients with HFpEF albeit similarly to both control [25] and HFrEF subjects [17]. Meanwhile, in patients with a sudden worsening of their heart failure symptoms or signs (i.e. acutely decompensated), elevated resting total plasma NE in HFpEF patients has been shown to be similar to that of HFrEF patients at the time of hospitalization [26].

The heterogeneity in findings from studies that measure total plasma NE in patients with HFpEF is likely due to a combination of factors. Firstly, total plasma NE can be a crude, inaccurate, and unreliable measure of global SNS activity due to numerous technical and biological limitations. Plasma levels of NE depend on both NE release and clearance, the latter is influenced by numerous factors such as renal function [14]. Indeed, in other pathological conditions such as hypertension or obesity where sympathetic overactivity has been demonstrated using direct microneurography, total plasma NE level can be normal [14]. Such a finding has also been observed in HFpEF [24].

Secondly, differing HFpEF disease characteristics (e.g., historical differences in threshold values defining “preserved” ejection fraction, underlying ischemia versus no ischemia, severity, trajectory), age, sex, individual co-morbidities (e.g., hypertension, obesity, diabetes mellitus), medication regimens, and other factors are potential confounders of total plasma NE (and indeed other measures of SNS activity) (Table 1). Therefore, a limitation of many studies is the inability to distinguish whether HFpEF itself, or some other factor, is responsible for changes in SNS activity. Finally, there is increasing recognition that HFpEF may represent a heterogenous syndrome [27] and elevated sympathetic activity may only be present in certain HFpEF phenotypes. Indeed, a handful of studies have previously reported elevated NE levels in a subset of HFpEF patients [22, 28]. This highlights the need for further detailed characterization of HFpEF patients to identify if certain HFpEF phenotypes consistently exhibit sympathetic overactivity and to what extent and where this sympathetic activation occurs. Taken together, HFpEF patients demonstrate substantial variability in total plasma NE levels, such that normal (or low) plasma NE may not necessarily reflect the absence of sympathetic hyperactivity in this syndrome.

### Activity of post-ganglionic sympathetic nerves in HFpEF

One of the characteristic features of the SNS is that sympathetic outflow to individual organs is non-uniform and is differentially regulated in both healthy and diseased states [14, 15]. Therefore, measuring total plasma NE may not provide quantitative information on regional sympathetic activity. One technique for evaluating region-specific SNS activity in humans is via direct intraneural recording (microneurography) of sympathetic nerves. In humans, post-ganglionic efferent sympathetic nerve activity in muscle (MSNA), which is vasoconstrictor in function, can be directly recorded in peripheral nerves (typically the peroneal nerve) using a needle inserted percutaneously [29]. In contrast to other methods of evaluating SNS activity, microneurography allows sympathetic nerve activity to be recorded over reasonably long periods of time (e.g., hours) while also allowing rapid and dynamically modulated changes in sympathetic nerve activity to be assessed, such as that during exercise [29].

#### Resting muscle sympathetic nerve activity in HFpEF

Modest elevations of resting MSNA are apparent in some patients with HFpEF. Studies have previously reported elevated resting MSNA in HFpEF patients when analyzed as burst frequency (bursts/min) [24, 30, 31]. However, when differences in heart rate are accounted for by analyzing MSNA as burst incidence (bursts/100 heart beats), only Seravalle et al. [24] and Kataoka et al. [31] observed increases in HFpEF patients. Importantly, these studies compared MSNA in HFpEF patients with healthy control participants. It is important to note that patients with HFpEF have co-morbidities so it remains unclear whether detected elevations of MSNA in HFpEF patients are intrinsic to the HFpEF syndrome or an epiphenomenon of its co-morbidities. In this context, preliminary data from conference proceedings indicate increased resting MSNA burst frequency [32, 33], but not burst incidence [34], in HFpEF patients when compared to co-morbid controls. Interestingly, resting MSNA may not necessarily relate to the type of heart failure as categorized by LVEF. For example, using spline modeling, Badrov et al. [35] demonstrated that LVEF only accounted for 9.8% of MSNA variance in heart failure patients and that MSNA was independent of LVEF once the latter rose above 21%. In contrast, Seravalle et al. [24] suggest that HFpEF is characterized by intermediate elevations of resting MSNA (higher than healthy controls but lower than heart failure with mid-range ejection fraction and HFrEF patients) while also reporting an inverse relationship between MSNA and LVEF when their study group was analyzed as a whole. Overall, current data on MSNA in HFpEF patients is limited and suggests that resting MSNA may only be moderately elevated in a subset of HFpEF patients. Early data suggests that one such group may be obese females with HFpEF [36]. However, the phenotype(s) of HFpEF patients with increased resting MSNA remains to be clearly defined.

#### Muscle sympathetic nerve activity during exercise in HFpEF

Exercise intolerance in HFpEF may be contributed, in part, by abnormalities in skeletal muscle morphology and function (skeletal myopathy) [4]. For example, HFpEF patients exhibit impaired skeletal muscle blood flow and vasodilation during exercise that is unrelated to changes in central hemodynamic variables such as cardiac output [37–40]. Whether this is due to excess MSNA remains to be directly proven since the current scant data has evaluated the response of MSNA to exercise in isolation (without concurrent assessment of skeletal muscle blood flow). In this context, current preliminary data shows that during *dynamic* (one-leg cycling) exercise, MSNA increases in HFpEF patients whereas MSNA is either unchanged in co-morbid controls or decreases in healthy controls [32, 33]. Similarly, during *static* exercise, preliminary data indicates that HFpEF patients have greater MSNA than co-morbid controls and healthy controls [34], although this is at odds with other preliminary findings, which show that static exercise elicits similar [41] or attenuated increases in MSNA [42] in HFpEF patients when compared to healthy control participants. Note that while the majority of these preliminary findings provide a mechanistic explanation for the diminished vasodilatory capacity of the skeletal muscle in HFpEF, impaired functional sympatholysis (ability of blood vessels to oppose sympathetically-mediated vasoconstriction) has also been reported in emerging data from HFpEF patients [43, 44].

### Indirect estimates of regional sympathetic nerve activity in HFpEF

MSNA itself is not always representative of efferent sympathetic nerve activity to other organs [14, 15]. While microneurography is considered the gold standard and preferred approach for assessing sympathetic nerve activity owing to its direct nature, intraneural recordings of efferent sympathetic nerve activity to internal organs, such as the heart or kidney, are not feasible in humans (but has been performed in animal models) due to the limited accessibility of these nerves. As such, indirect techniques have been used to evaluate regional sympathetic nerve activity. The following section discusses the studies that have examined SNS activity at the levels of the heart, kidney, and the splanchnic circulation.

#### Cardiac sympathetic nerve activity in HFpEF

Since direct recordings of cardiac sympathetic nerve activity are not possible in humans, invasive measurement of NE spillover can be used to infer cardiac sympathetic outflow. Unfortunately, no current human data on cardiac-specific NE spillover exists for HFpEF. Nonetheless, the transcardiac NE gradient (this is not cardiac NE spillover as it does not account for the extraction of arterial norepinephrine and alterations in coronary blood flow) is increased in HFpEF patients compared to healthy subjects [45]. However, on multivariable analysis, only systolic blood pressure (and not a diagnosis of HFpEF) was associated with the transcardiac NE gradient, suggesting that hemodynamic factors probably play a key role in determining the NE gradient in HFpEF.

In addition to NE spillover, the visualization and quantification of radiolabeled NE analogues can be used to infer the anatomy and function of the cardiac SNS at the post-ganglionic, synaptic, and post-synaptic interface (i.e. neuroeffector junction) (cardiac neurotransmission imaging). In cardiac [^123^I] *meta*-iodobenzylguanidine (*m*IBG) imaging, parameters such as late (delayed) heart-to-mediastinum ratio (H/M) and washout rate (WR) are commonly evaluated with the following assumptions made: (i) late H/M provides combined information on pre-synaptic uptake, storage and release of NE and (ii) WR represents neuronal integrity or sympathetic tone (presumably due to uptake-1 function) [46]. Together, it is assumed that low late H/M and high WR reflects increased sympathetic activity. However, this technique (and [^11^C]hydroxyephedrine imaging described below) cannot delineate the mechanistic cause of imaging defects, which could be due to (i) increased systemic NE competing for uptake-1 sites, (ii) increased post-ganglionic release of NE into the synaptic cleft which competes for uptake-1, (iii) downregulation or inhibition of uptake-1, and/or (iv) anatomic denervation (loss of nerve density) [47]. As such, “dysfunction” will be used to describe *m*IBG findings herein, reflecting the inability to distinguish between anatomical denervation and abnormal synaptic transmission.

In this context, compared to unmatched control participants without heart failure, HFpEF patients have lower H/M and higher WR on cardiac *m*IBG imaging [48]. However, compared to HFrEF patients, those with HFpEF appear to have less cardiac sympathetic dysfunction, as indicated by their higher late H/M and lower WR [18, 49]. Additionally, HFpEF patients with lower late H/M and/or higher WR (i.e. greater cardiac sympathetic dysfunction) have more severe symptoms, reduced exercise capacity, and are at greater risk of future cardiovascular events [48–52]. Importantly, caution should be exercised in generalizing these findings to the wider HFpEF population as the majority of these cardiac *m*IBG imaging studies were small scale, single center, and frequently included both primary and secondary (i.e. HFpEF caused by an underlying condition such as an arrhythmia or valvular disease) HFpEF patients. A number of these studies were also undertaken on acutely decompensated heart failure patients in the convalescent (clinically stable) stage of their hospitalizations where it has been shown, based on *m*IBG imaging parameters, that rapid decongestion using conventional diuretic therapy causes an increase in cardiac sympathetic dysfunction [53].

Cardiac sympathetic dysfunction has also been observed in HFpEF patients using [^11^C]hydroxyephedrine (HED) imaging. HED has similar properties to *m*IBG with its retention assumed to reflect pre-synaptic neuronal activity from uptake through to vesicular packaging and release. As such, HED uptake, which is commonly reported as a retention index (RI), is closely correlated with late H/M on *m*IBG imaging [54]. Compared to age-matched controls without heart failure, HFpEF patients have a lower HED RI [20, 55]. HED RI also decreases (i.e. more sympathetic dysfunction) with the severity of diastolic dysfunction in HFpEF [20]. Of note, these studies may have inadvertently included heart failure with mid-range ejection fraction and/or improved ejection fraction patients as they used a LVEF threshold of 40% to define HFpEF and they included both primary and secondary HFpEF patients, some of whom also had atrial fibrillation. Indeed, the median LVEF in these studies progressively decreased with the severity of diastolic dysfunction (e.g., median LVEF of 67% in non-heart failure control subjects versus 45% in “HFpEF” patients with grade 2–3 diastolic dysfunction) and/or the absence of coronary artery disease. Taken together, cardiac neurotransmission imaging studies suggest that HFpEF patients have modest “dysfunction” of the cardiac sympathetic neuroeffector junction, although not to the same extent as HFrEF patients.

#### Renal and splanchnic sympathetic nerve activity in HFpEF

The inhibition of sympathetic nerve signaling, achieved mechanically via surgical transection or radiofrequency ablation of sympathetic nerves (herein referred to as “denervation”), is one method of inferring region-specific sympathetic activity. It must be noted that given the inability to directly record from nerves innervating internal organs, there is a lack of direct evidence that sympathetic nerve activity to/from these specific organs is elevated in HFpEF. Similarly, while there is no evidence that the spillover of NE from these organs is elevated in HFpEF, it has not deterred the denervation of sympathetic nerves supplying the kidneys and splanchnic circulation in HFpEF patients.

Renal denervation has been performed in a small number of HFpEF patients. In an analysis by Kresoja et al. [56], patients with resistant hypertension who were retrospectively identified as having HFpEF at the time of catheter-based renal denervation had improved heart failure symptoms and echocardiographic parameters of diastolic function following the procedure. These improvements were independent of reductions in blood pressure but the precise contributions of renal denervation to the improvements reported were not entirely clear as the study was non-randomized, did not have a sham-control group, and medication adherence following renal denervation was not monitored. The findings of Kresoja et al. [56] also contrast with those of an earlier albeit underpowered (due to recruitment difficulties), randomized, prospective, open-controlled trial in HFpEF patients showing transcatheter renal denervation had no effect on any of the primary efficacy endpoints (heart failure symptoms, exercise capacity, quality of life, and cardiac imaging parameters of diastolic function) [28]. While the small study size may have contributed to these negative findings, renal denervation studies are also frequently limited by their inability to determine procedural success (or the “completeness” of denervation) [57]. For example, renal NE spillover rate is infrequently assessed. Moreover, renal nerves can re-innervate the kidney following ablation [58], and it can be difficult to determine if the effects of renal denervation are due to disrupted afferent sensory versus efferent sympathetic signaling [59].

The splanchnic circulation is a highly compliant vascular bed that receives 25–30% of cardiac output under normal conditions and is a major reservoir of intravascular blood volume. Under rich sympathetic innervation, the splanchnic circulation can alter cardiac filling pressures by regulating the distribution of blood between the peripheral and central vascular compartments [60]. Therefore, the untested assumption is that sympathetic activity to the splanchnic circulation is elevated in HFpEF patients. In this regard, the greater splanchnic nerve (GSN), which is the major carrier of pre-ganglionic sympathetic nerves to the splanchnic vascular bed, has recently been identified as a potential target in the management of HFpEF. In an anesthetized HFpEF patient, acute percutaneous GSN stimulation elicited transient increases in central venous pressure, pulmonary artery pressure, and left atrial pressure [61]. These increases in central and cardiac hemodynamic parameters are usually unfavorable for HFpEF patients given that they are associated with exercise intolerance [62]; however, exercise capacity was not assessed in this anesthetized GSN stimulation study. Conversely, when the right GSN is interrupted in HFpEF patients via surgical [63] or percutaneous radiofrequency ablation [64–66], cardiac filling pressure (as inferred by pulmonary capillary wedge pressure) during exercise is reduced and heart failure symptoms, exercise capacity, and quality of life are improved. It is important to note that these are early findings from small, open-label trials with a follow-up period of up to 12 months and that randomized, controlled trials are currently in progress to confirm these findings.

### Global pharmacological sympathoinhibition in HFpEF

Aside from mechanical sympathetic denervation, activity of the SNS can also be inferred by measuring a response following acute pharmacological blockade of receptors involved in adrenergic transmission. Broadly speaking, sympathoinhibitory drugs can act at three different levels: (i) peripherally at the post-synaptic (effector) level as α_1_- (e.g., phentolamine) or β-adrenergic (e.g., propranolol) receptor antagonists, (ii) within autonomic ganglia via blockade of nicotinic acetylcholine receptors (e.g., hexamethonium), and (iii) centrally (within the brainstem) as α_2_-adrenergic agonists (e.g., clonidine). In all cases, the confounding effects of non-specific receptor antagonism/agonism and the completeness of receptor antagonism/agonism should be considered.

#### Use of β-blockers in HFpEF

As alluded to previously, there is a lack of concrete evidence that cardiac sympathetic activity is elevated in HFpEF patients. Despite this, β-blockers are used empirically and with high prevalence in HFpEF, with 50–80% of HFpEF patients receiving β-blocker therapy [67]. While prognostic benefit may be derived from the use of β-blockers in the management of certain co-morbidities that may be associated with HFpEF (e.g., symptomatic coronary artery disease, recent myocardial infarction, atrial fibrillation) [68, 69], the high propensity for its use in HFpEF is likely due to clinicians’ perceived benefit derived from HFrEF studies. In this regard, some data suggest that β-blockers are effective at reducing heart failure hospitalization and mortality in a phenogroup of HFpEF patients characterized by higher body mass index, greater burden of co-morbidities, more severe heart failure symptoms, and higher B-type natriuretic peptide levels [70].

However, when taken as a whole in meta-analyses, the use of β-blockers in HFpEF has shown mostly neutral or dubious benefits. In a Cochrane review, the potential benefit of β-blockers in HFpEF (when compared to placebo or no treatment) on cardiovascular mortality was abolished when sensitivity analysis was performed to include only studies deemed to be at low overall risk of bias. Additionally, the effects of β-blockers in HFpEF on heart failure hospitalization and quality of life were uncertain [71]. These findings were similar to a meta-analysis of individual patient data from randomized controlled trials, which showed that β-blocker use in HFpEF did not improve LVEF or reduce cardiovascular hospitalization or mortality [72]. Concerningly, recent analysis of registry data consisting of over 360,000 HFpEF patients indicated that while β-blocker use lacked survival benefit, it increased the risk of heart failure hospitalization, particularly in those with LVEF > 60% [73]. It is important to note that β-blockers vary in their pharmacological properties and may therefore have differing effects on outcomes [74]. When β-blockers are considered as a single group of medications, as they are in meta-analyses, the effects of dose, duration, and class types of β-blockers (such as those with peripheral vasodilator action, which might reduce cardiac afterload) are lost.

While clinical trials frequently focus on hospitalization and mortality as outcomes, the impact of β-blockers on other outcomes such as exercise capacity is arguably of greater relevance to the day-to-day life of the individual HFpEF patient. In this context, β-blockers are known to compromise the determinants of cardiac output and may therefore contribute to exercise intolerance. For example, β-blockers can cause chronotropic incompetence [75, 76] and impair both ventricular relaxation [77, 78] and contraction [74, 79]. However, surprisingly little attention has been given to whether β-blockers exert similar, potentially deleterious, effects on exercise hemodynamics in the context of HFpEF, and is an area that requires further elucidation. In keeping with this, meta-analysis of the limited number of randomized controlled trials available suggests that β-blockers do not improve exercise capacity in HFpEF [80]. Moreover, when β-blocker therapy is withdrawn (for 2 weeks) in HFpEF patients with known chronotropic incompetence, peak exercise heart rate and exercise capacity are improved, along with surrogate markers for quality of life and resting left ventricular filling pressure [76].

Finally, recent observational data shows that female, but not male, HFpEF patients taking β-blockers have higher B-type natriuretic peptide levels [81]. Such a finding is consistent with the increasing recognition that sex differences likely play an important role in the pathophysiology of HFpEF [82]. However, definitive sex-specific data on the SNS in HFpEF are currently lacking and is an area that requires further investigation and detailed consideration.

## Conclusion

**Fig. 1:**
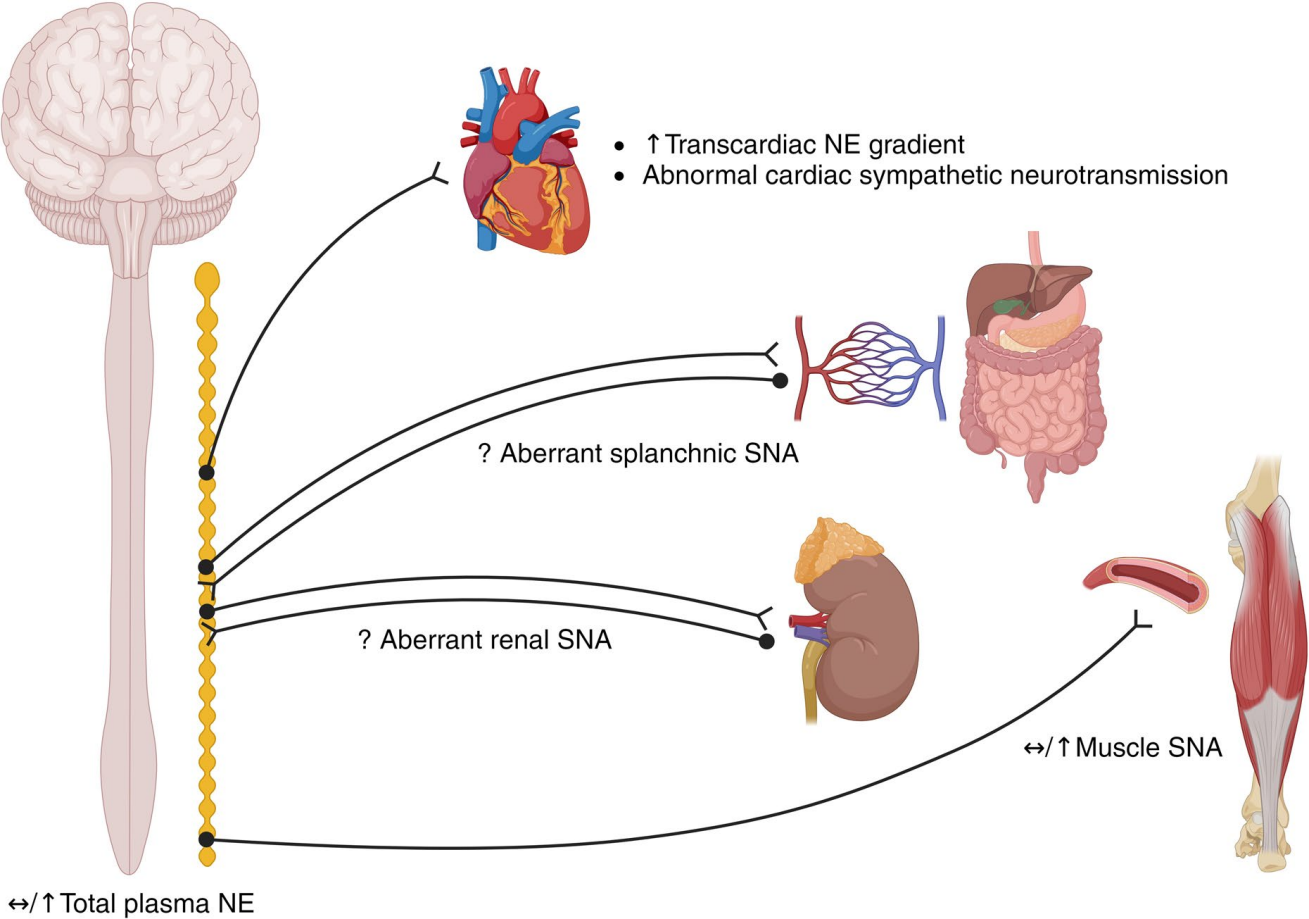
Activity of the sympathetic nervous system in HFpEF under resting conditions. NE, norepinephrine; SNA, sympathetic nerve activity. (Created with Biorender.com)

The current limited available body of evidence suggests that if present, SNS abnormalities are modest in HFpEF (Fig. 1) and may be predominantly an epiphenomenon of the co-morbidities associated with the syndrome. While moderate increases in resting levels of MSNA are apparent in HFpEF patients, this only appears so when compared to healthy (and not co-morbid) control participants. At the level of the heart, some evidence exists for dysfunctional sympathetic signaling but no clear evidence for elevated cardiac sympathetic nerve activity. The lack of a compelling prognostic benefit with use of β-blockers in HFpEF patients also supports this line of reasoning. Additionally, there is no evidence for elevated sympathetic drive to/from the kidneys or splanchnic circulation, yet interventional (denervation) studies have already been performed in HFpEF patients. Taken together, we conclude that there is minimal evidence for elevated resting sympathetic nerve activity in HFpEF, although how the SNS responds during exertion and how it might regulate the determinants of exercise capacity in HFpEF remain unknown and require urgent investigation.

## Data Availability

No datasets were generated or analysed during the current study.
